# Supplementary figures and images for: Genome-wide analysis of basic helix-loop-helix transcription factors in papaya (Carica papaya L.)
Source: PeerJ. 2020 Jul 3;8:e9319. doi: 10.7717/peerj.9319 (PMC7341539; doi:10.7717/peerj.9319)

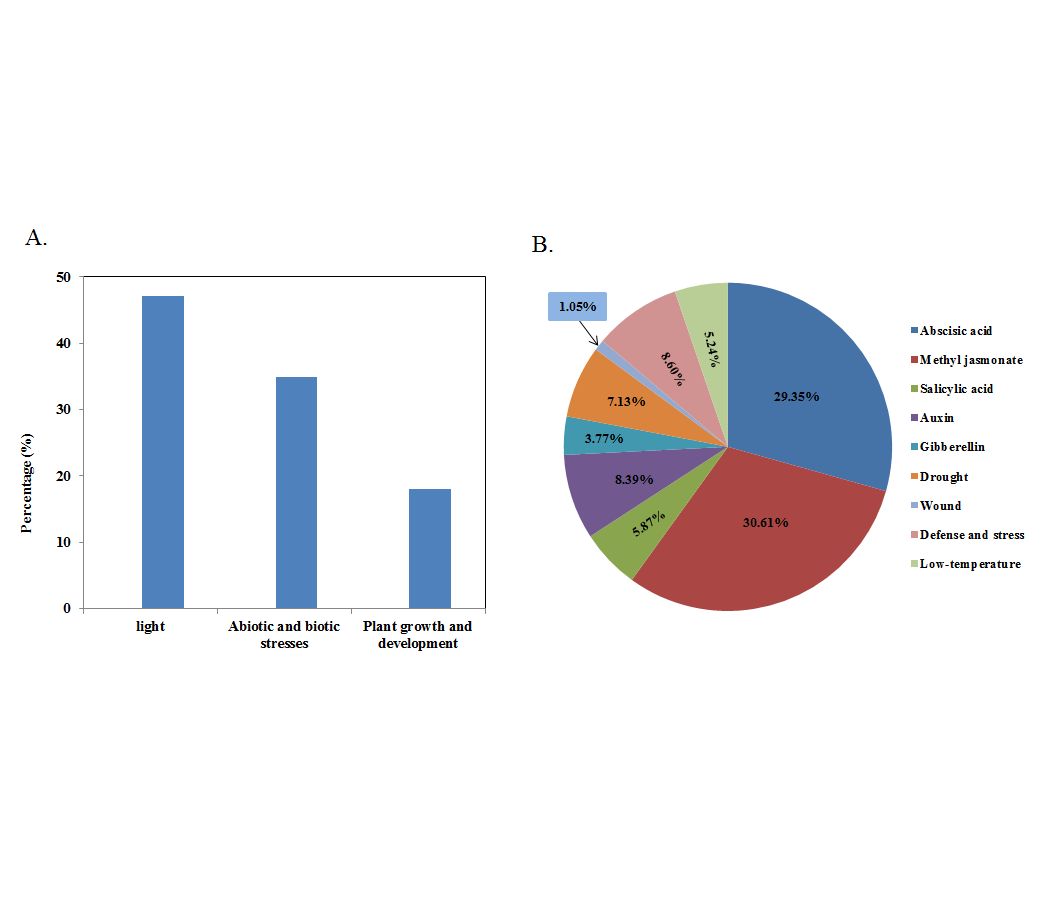

Supplement: Supplemental Information 10 — (A) Percentage of total cis-acting elements in the promoter region of At bHLH genes. (B) The percentage of each cis-acting element in the abiotic and biotic stresses categories. [file peerj-08-9319-s010.png]
